# Evaluating cell type deconvolution in FFPE breast tissue: application to benign breast disease

**DOI:** 10.1093/nargab/lqae098

**Published:** 2024-08-06

**Authors:** Yuanhang Liu, Robert A Vierkant, Aditya Bhagwate, William A Jons, Melody L Stallings-Mann, Bryan M McCauley, Jodi M Carter, Melissa T Stephens, Michael E Pfrender, Laurie E Littlepage, Derek C Radisky, Julie M Cunningham, Amy C Degnim, Stacey J Winham, Chen Wang

**Affiliations:** Department of Quantitative Health Sciences, Mayo Clinic, Rochester, MN 55905, USA; Department of Quantitative Health Sciences, Mayo Clinic, Rochester, MN 55905, USA; Department of Quantitative Health Sciences, Mayo Clinic, Rochester, MN 55905, USA; Department of Quantitative Health Sciences, Mayo Clinic, Rochester, MN 55905, USA; Biomedical Engineering and Physiology Graduate Program, Mayo Clinic Graduate School of Biomedical Sciences, Rochester, MN 55905, USA; Department of Cancer Biology, Mayo Clinic, 4500 San Pablo Road, Jacksonville, FL 32224, USA; Department of Quantitative Health Sciences, Mayo Clinic, Rochester, MN 55905, USA; Department of Laboratory Medicine & Pathology, University of Alberta, Edmonton, AB T6G 2R3, Canada; Genomics and Bioinformatics Core Facility, University of Notre Dame, Notre Dame, IN 46556, USA; Department of Biological Sciences, 109B Galvin Life Science Center, University of Notre Dame, Notre Dame, IN 46556, USA; Department of Chemistry and Biochemistry, Harper Cancer Research Institute, University of Notre Dame, Notre Dame, IN 46556, USA; Department of Cancer Biology, Mayo Clinic, 4500 San Pablo Road, Jacksonville, FL 32224, USA; Department of Laboratory Medicine and Pathology, Mayo Clinic, 200 1st Street SW, Rochester, MN 55905, USA; Department of Surgery, Mayo Clinic, 200 1st Street SW, Rochester, MN 55905, USA; Department of Quantitative Health Sciences, Mayo Clinic, Rochester, MN 55905, USA; Department of Quantitative Health Sciences, Mayo Clinic, Rochester, MN 55905, USA

## Abstract

Transcriptome profiling using RNA sequencing (RNA-seq) of bulk formalin-fixed paraffin-embedded (FFPE) tissue blocks is a standard method in biomedical research. However, when used on tissues with diverse cell type compositions, it yields averaged gene expression profiles, complicating biomarker identification due to variations in cell proportions. To address the need for optimized strategies for defining individual cell type compositions from bulk FFPE samples, we constructed single-cell RNA-seq reference data for breast tissue and tested cell type deconvolution methods. Initial simulation experiments showed similar performances across multiple commonly used deconvolution methods. However, the introduction of FFPE artifacts significantly impacted their performances, with a root mean squared error (RMSE) ranging between 0.04 and 0.17. Scaden, a deep learning-based method, consistently outperformed the others, demonstrating robustness against FFPE artifacts. Testing these methods on our 62-sample RNA-seq benign breast disease cohort in which cell type composition was estimated using digital pathology approaches, we found that pre-filtering of the reference data enhanced the accuracy of most methods, realizing up to a 32% reduction in RMSE. To support further research efforts in this domain, we introduce SCdeconR, an R package designed for streamlined cell type deconvolution assessments and downstream analyses.

## Introduction

Benign breast disease (BBD) is viewed as a potential precursor in the development of breast cancer (1), and genomic and transcriptomic aberrations identified at the time of BBD diagnosis can provide biomarkers for future breast cancer risk. Several studies have evaluated the significance of those aberrations, mainly focusing on genomic alterations (2–4). Given the long-term follow-up required for these investigations, the specimens typically come from archival sources. The formalin-fixed paraffin-embedded (FFPE) archival process is routinely used for clinical biospecimens for diagnosis and long-term storage, and has many advantages, such as room temperature stability and suitability for subsequent immunohistochemical (IHC) analyses (5). However, successful transcriptomic and genomic measurements from FFPE tissues have remained challenging, due to low quality of RNA and DNA extracted from FFPE tissue blocks and nucleotide artifacts introduced by the FFPE process (6,7).

Transcriptome profiling using RNA sequencing (RNA-seq) of bulk FFPE tissue blocks is a standard method in biomedical research (8–10). However, profiling heterogeneous samples yields an averaged expression, merging diverse cell types and their distinct states (11). Consequently, downstream analyses, such as biomarker discovery, will likely be confounded by differences in cell proportions across specimens. BBD tissue is inherently complex, consisting of multiple cellular components, including epithelial cells, stromal cells and immune cells. Our previous work has demonstrated that changes in these cellular compositions, particularly the involution of terminal duct lobular units, are associated with breast cancer risk (12). Deconvolution of this cell type composition in BBD tissue is crucial to identify transcriptomic alternations linked to future breast cancer risk. In this study, our primary focus is to identify the most effective deconvolution strategies for archival breast tissue samples. Nonetheless, similar challenges are present in other tissue types, and we anticipate that findings from our research can be adapted and proven beneficial to other tissue types.

Many computational methods have been developed over the last decade to address this issue. Traditional marker gene-based deconvolution algorithms predominantly rely on gene expression profiles of signature or marker genes for all cell types found in the bulk tissue. With the advent of single-cell RNA-seq (scRNA-seq) technology, it is now possible to achieve gene expression profiling at a single-cell resolution, leading to the development of deconvolution algorithms directly based on scRNA-seq data. However, due to the varying sensitivity of single-cell protocols in detecting distinct cell types, there has been a notable gap in efforts to create a comprehensive single-cell reference by integrating data from different protocols specifically for breast tissue.

Existing deconvolution methods can be roughly grouped into three main categories according to their input requirements and underlying statistical models (13–22): (i) marker gene-based regression methods (MG-based), such as CIBERSORT (19), OLS (13) and FARDEEP (16); (ii) scRNA-seq transcriptome-based conventional statistical methods (SC-based), such as MuSiC (22), SCDC (15) and CIBERSORTx (20); and (iii) scRNA-seq transcriptome-based deep learning methods (DL-based), such as Scaden (17) and scTAPE (14). Several benchmarking studies have been conducted to evaluate the performance of different deconvolution methods (11,23,24), yet most are tailored for fresh-frozen (FFzn) samples and often neglect particular challenges associated with archival tissues. We have previously demonstrated that quality of RNA-seq data generated from FFPE tissues is highly dependent on RNA qubit values. FFPE replicates yield higher overall false positive rate (lower reproducibility) compared to FFzn replicates, especially for lowly expressed genes (7). The FFPE processing and subsequent tissue storage procedures are known to result in highly degraded RNAs, thereby impairing accurate gene expression quantification through RNA-seq. The implications of those challenges associated with FFPE samples on cell type deconvolution analysis remain unclear, and it is uncertain whether additional considerations are necessary for accurate cell type deconvolution. However, prior benchmarking studies evaluating various deconvolution algorithms often overlooked these specific challenges inherent to FFPE samples. For example, Avila Cobos *et al.* (23) evaluated how data transformation, pre-processing and marker selection strategies impact deconvolution results. To benchmark different deconvolution methods, they generated simulated datasets and also utilized real data, where cell type proportions were measured by flow cytometry. However, the applicability of flow cytometry to FFPE tissue is challenging due to the fixation process that can negatively impact cell integrity and the fluorescent properties of markers (25). Alternative methods are needed to derive reliable benchmarking data for deconvolution processes when applied to archival tissues.

To fill the gaps in RNA-seq deconvolution assessments for FFPE tissue, we designed a comprehensive simulation study, complemented by real dataset evaluations in the context of breast tissue. We first constructed scRNA-seq reference data for breast tissue by integrating data from two single-cell protocols. Computational simulations were then conducted to evaluate RNA-seq deconvolution methods under three distinct scenarios: (i) baseline; (ii) incomplete reference set; and (iii) FFPE artifacts. We assessed these methods using conventional metrics such as root mean squared error (RMSE) and Spearman correlation coefficient (SCC), as well as dissected metrics like bias and variance. We then examined the performance of deconvolution methods using digital annotations of cell type proportions in BBD FFPE samples. These annotations were generated in-house through CaseViewer, an artificial intelligence-powered tool for digital cell type annotation. Finally, we validated the applicability of our optimized deconvolution strategy on FFzn samples using pathology annotations from the GTEx breast tissue cohort. To facilitate future studies involving FFPE samples from all tissue types, we built an R package, SCdeconR, including a simulation framework and an inference module. It is currently available from CRAN (https://CRAN.R-project.org/package=SCdeconR) and GitHub (https://github.com/Liuy12/SCdeconR).

Several toolkits have been developed previously for cell type deconvolution analysis based on RNA-seq data, such as Immunedeconv (26) and ADAPTS (27). Immunedeconv provides a unified framework that focuses on estimating immune cell fractions from bulk RNA-seq data. However, it is pertinent to note that the utility of Immunedeconv may be constrained in the context of identifying non-immune cell types. Notably, the efficacy of algorithms included in Immunedeconv is dependent upon the signature matrices curated by each respective method. There are large variabilities in terms of the collection and granularity of cell types among those methods. For instance, MCP-counter (28) only estimates cell proportions for four cell types, including neutrophils, cancer-associated fibroblasts, endothelial cells and T cells. As a contrast, xCell (29) estimates cell proportions for 35 cell types with high granularity, including different subtypes of immune cells. This results in inconsistency in cell type outputs among methods. ADAPTS is a tool that emphasizes optimizing cell signature matrix construction used for deconvolution, and includes only four deconvolution algorithms, DCQ (30), CIBERSORT (19), DeconRNASeq (31) and Admixture (32). In contrast, SCdeconR offers more functionality than a mere wrapper for deconvolution methods, exhibiting numerous advantages over existing toolkits. First, it features a dedicated module for constructing single-cell reference data from different sources. Second, it facilitates the generation of reproducible simulated experiments for future evaluation studies, allowing for comparisons of various deconvolution methods under a variety of scenarios that can be tailored to expected data characteristics. Third, SCdeconR provides a unified and generalizable interface for cell type deconvolution for bulk RNA-seq data, alongside streamlined functionality for downstream analyses, including differential expression analysis (DEA) and gene set enrichment analysis (GSEA). In this study, we aim not only to address the current limitations of RNA-seq deconvolution for FFPE tissues but also to provide a robust tool that will further the field’s ability to accurately interpret archival as well as FFzn samples. By integrating cutting-edge methodologies and real-world data applications, we endeavor to elevate the standards of genomic studies, ensuring that they remain both precise and applicable across a spectrum of tissue types.

## Materials and methods

### Study design

**Figure 1.**
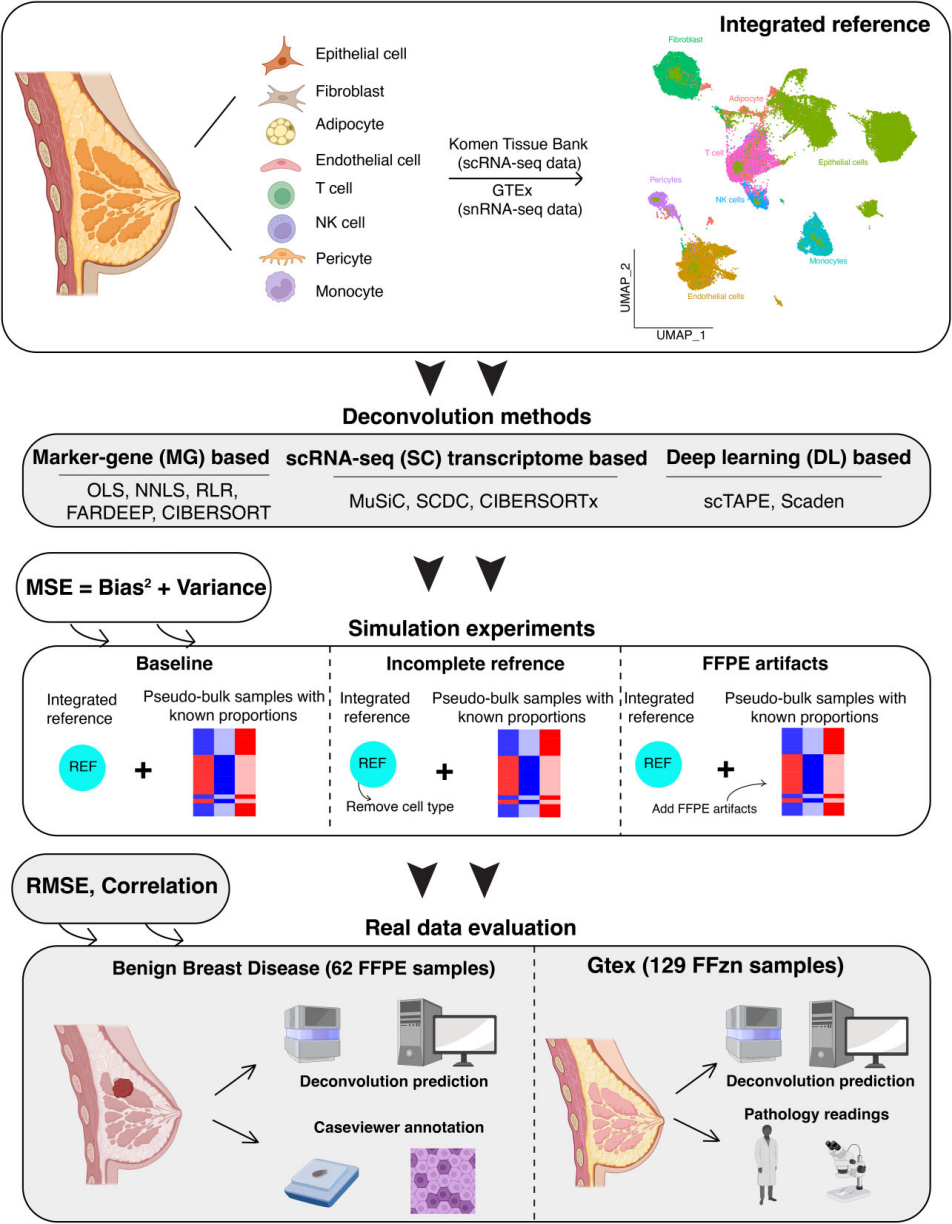
Overview of the study framework. The diagram illustrates three primary phases of our research: construction of scRNA-seq reference data for breast tissue; comparative analysis of deconvolution methods through simulation experiments; and evaluation of these methods on real data, encompassing both FFPE and FFzn samples (created with BioRender.com).

Initially, we constructed a single-cell reference for female breast tissue using data from two independent studies involving different protocols (Figure 1). Across these studies, eight cell types were characterized. We subsequently curated 10 deconvolution methods spanning the three categories (MB-based, SC-based, DL-based). Using the previously assembled single-cell reference data, we created artificial bulk RNA-seq samples through random sampling. We then conducted three types of simulation experiments to assess the deconvolution methods in terms of (i) baseline performance under optimal conditions, (ii) the effects of missing cell types in the reference data and (iii) the impact of FFPE artifacts. We extended our evaluation to in-house generated real data, which included imaging-based digital annotation for a cohort of 62 BBD samples, and manually interpreted reports from pathology impression for a cohort of 192 FFzn samples from the GTEx project. We evaluated deconvolution methods on both overall performance across cell types and their performance within individual cell types.

### Generation of integrated reference for breast tissue

We selected two scRNA-seq studies focused on female breast tissue. The first, by Bhat-Nakshatri’s group, performed scRNA-seq on 11 normal adult female breast samples sourced from the Komen Tissue Bank (33). The raw data (cell-ranger output) were accessed from Gene Expression Omnibus (GEO; GSE164898). The second, by the GTEx Consortium, performed single-nucleus RNA-seq (snRNA-seq) on three normal adult female breast samples from their cohort (34). Raw data (.h5ad) were downloaded from the GTEx single-cell portal. Both datasets were subjected to rigorous quality control (QC) to remove low-quality cells. Criteria for removal included cells that had fewer than 200 features or a mitochondrial concentration exceeding 40%. Raw count data were then normalized using SCTransform v2 (35). Samples from the two cohorts were integrated using the Harmony algorithm (36) based on the first 30 principal components. Cell type annotations from the original studies guided the labeling of the merged cell clusters. Overall, eight cell types were characterized from the two studies: epithelial cells, fibroblasts, adipocytes, endothelial cells, T cells, natural killer (NK) cells, pericytes and monocytes.

### Cell type deconvolution algorithms

The cell type deconvolution problem for bulk tissue-derived RNA-seq data can be formulated as


(1) \begin{eqnarray*}B = {S^{\rm T}}P,\end{eqnarray*}

where *B* represents a bulk RNA-seq gene expression matrix of dimensions *i* × *j*, where rows represent genes and columns represent samples. *S* is a cell type-specific signature matrix of dimensions *k* × *i*, with rows representing cell types and columns representing genes. *P* is cell type proportion matrix of dimensions *k* × *j*, with rows representing cell types and columns representing samples. The goal is to determine *P* using *S* and *B* as input. We curated 10 deconvolution methods from three different categories: (i) MG-based; (ii) SC-based; and (iii) DL-based.

*MG-based*: We included two conventional least squares-based methods, ordinary least squares (OLS) (13) and non-negative least squares (NNLS) (18). Compared to OLS, the NNLS algorithm adds one extra constraint that requires all coefficients to be nonnegative. We implemented the OLS algorithm in R, and utilized the nnls package (version 1.4) from CRAN for NNLS. CIBERSORT is a deconvolution algorithm based on support vector regression. We obtained the R wrapper script for CIBERSORT from the authors (19). Two robust regression-based methods were also included: FARDEEP (version 1.0.1) (16) and RLR (21). RLR was implemented in R based on the MASS package (version 7.3).

*SC-based*: CIBERSORTx is a recent variant of CIBERSORT that directly utilizes scRNA-seq data as input to generate a gene expression signature matrix (20). It also provides functionality for batch normalization to enhance robustness of deconvolution. For simulation and real-data evaluation using CIBERSORTx, we uploaded required input files to the web portal (https://cibersortx.stanford.edu/). MuSiC is a deconvolution algorithm that uses multi-subject scRNA-seq datasets and computes weights based on cross-subject consistency (version 0.2.0) (22). Similarly, SCDC is another deconvolution algorithm that integrates multiple scRNA-seq references and weights references based on their similarity with provided bulk RNA-seq data (version 0.0.0.9000) (15).

*DL-based*: Scaden is a recently developed method based on ensemble of three independent multilayer neural networks (17). Python implementation of Scaden (version 1.1.2) was used in this study via the R package reticulate (version 1.20). scTAPE is another deep learning-based deconvolution method utilizing autoencoders (14). Python implementation of scTAPE (version 1.1.2) was used within R via the reticulate package.

### Simulation framework

Three types of simulation experiments were performed to examine different deconvolution methods regarding (i) baseline performance in an optimal scenario, (ii) sensitivity to missing cell types from the reference and (iii) robustness to the introduction of FFPE artifacts. For the baseline experiment, we first split the constructed breast tissue scRNA-seq reference data into training and testing data with a split ratio of 50%. Then, we randomly simulated 50 vectors of cell type proportions (*p*). For each *p*, 10 artificial bulk RNA-seq samples were generated by randomly sampling from the training reference data according to simulated cell type proportions. This unique simulation design enables downstream evaluations based on metrics like bias and variance for a better understanding of source of prediction errors from each deconvolution method. A total of 500 (50 × 10) artificial bulk samples were generated using this procedure. Finally, the artificially generated bulk samples as well as the testing reference data were provided to the deconvolution methods for prediction. For incomplete reference experiments, similar procedures to the baseline experiments were performed to generate artificial bulk samples. To evaluate different methods on their performances regarding incomplete references, for each cell type, we artificially removed the gene expression data for that specific cell type from the testing reference data. Subsequently, cell type proportions were predicted using deconvolution methods with the simulated bulk data and ‘incomplete’ testing reference data. For FFPE artifact experiments, we first followed a similar procedure to the baseline experiment to generate artificial bulk RNA-seq samples. To introduce FFPE artifacts to the simulated bulk data, we built a generalized additive model to learn the relationship between FFPE artifacts and gene expression intensity using seven in-house generated paired FFPE and FFzn samples:


(2) \begin{eqnarray*}{\rm Model}({G_i} ):\;{y_i}\sim s\left({x_i} \right),\end{eqnarray*}

where *y_i_* indicates average absolute differences between FFPE–FFzn paired samples (FFPE artifacts) for gene *G*_*i*_and *x_i_* indicates average gene expression level in transcripts per million (TPM) for gene *G_i_*. For gene *G_i_* in sample *j*, FFPE artifacts were introduced to the above-generated artificial bulk data from Gaussian distribution *N* with mean estimated from Model(*G*_*i*_) and standard deviation of 0.5; *α* is a random direction indicator sampled from vector [−1, 1]:


(3) \begin{eqnarray*}G_{ij}^{{\rm FFPE}} = {G_{ij}} + \alpha \times N( {M( {{G_{ij}}} ),0.5} ).\end{eqnarray*}

For all three simulation experiments, the predicted cell type proportions for each deconvolution algorithms were compared to the known simulated cell type proportions. Three metrics were used for evaluation:


(4) \begin{eqnarray*}{\rm RMSE} = \sqrt{ \frac{{\mathop{\sum \nolimits_{i = 1}^n} \| {y_i} - \widehat {{y_i}} \|^2}}{n} },\end{eqnarray*}

where *n* represents number of samples simulated from the same proportion vector *p*, in this case 10. *y_i_* indicates known simulated cell type proportions. $\widehat {{y_i}}$ indicates predicted cell type proportions. Bias is calculated as


(5) \begin{eqnarray*}{\rm bias} = \mathop \sum \limits_{i = 1}^n \widehat {{y_i}}/n - y,\end{eqnarray*}

and variance is calculated as


(6) \begin{eqnarray*}{\rm variance} = \mathop \sum \limits_{i = 1}^n {\left( {\widehat {{y_i}} - \mathop \sum \limits_{i = 1}^n \widehat {{y_i}}/n} \right)^2}/n.\end{eqnarray*}

The three metrics are related by


(7) \begin{eqnarray*}{\rm MSE} = {{\rm bias}^2} + {\rm variance}.\end{eqnarray*}

The simulation framework is built upon a previous benchmarking study (23), and available within SCdeconR package from CRAN (https://CRAN.R-project.org/package=SCdeconR) and GitHub (https://github.com/Liuy12/SCdeconR).

### Cell type digital annotation using CaseViewer

Biopsies mounted on microscopic slides were stained with hematoxylin and eosin (H&E). The slides were digitally scanned at 20× using the Pannoramic 250 Flash III (3DHISTECH Ltd, Budapest, Hungary) slide scanner. The tissues on these slides were segmented into three distinct categories: epithelia, stroma and adipose. This segmentation was accomplished using the trainable pattern recognition model, PatternQuant, embedded within the CaseViewer software (version 2.3) by 3DHISTECH Ltd. To effectively train the algorithm, multiple exemplars of each tissue type were provided. The reliability of this trained model was subsequently validated on a test batch of 12 images. After classification, the CellQuant module was used to count the number of nuclei present within each segmented region. Nuclear identification was based on stain intensity and morphological features. Lastly, the percentage distribution for each of the three cell types was computed by dividing the nuclei count of each cell type by the aggregate count of detected nuclei across all segments.

### IHC analysis

Biopsies mounted on microscopic slides were stained with anti-CD45 (Abcam, ab10559) at 1:800. Slides were digitally scanned at 20× using the Pannoramic 250 Flash III (3DHISTECH Ltd, Budapest, Hungary) slide scanner. Nuclei (positively and negatively stained) were identified using the nuclear recognition portion of CellQuant, embedded within the CaseViewer software (version 2.3) by 3DHISTECH Ltd. Nuclear identification was based on stain intensity and morphological features. The percentage distribution of positively stained nuclei was computed by dividing the positive nuclei count by the total nuclei count and converting to percentage.

### Evaluation using FFPE samples from the BBD cohort

RNA-seq was available for 130 FFPE samples for our BBD cohort study. A comprehensive QC process, as described previously (7), was employed to exclude low-quality samples. This resulted in a refined dataset consisting of 62 samples for the study. Cell type proportions based on number of nuclei were quantified using a combination of PatternQuant and CellQuant algorithms from CaseViewer. These quantifications served as the benchmark or ‘ground truth’ for assessing the performance of different deconvolution algorithms. To predict cell type proportions from bulk RNA-seq data using deconvolution algorithms, we first filtered out genes with average TPM lower than 4 from the bulk data, given their vulnerability to FFPE artifacts (7). Bulk RNA-seq data were normalized to TPM using the TMM method from edgeR (version 3.36.0) (37). scRNA-seq reference data were normalized using the *LogNormalize* function from Seurat (version 4.9.9) (38). Protocol differential genes between scRNA-seq and snRNA-seq reference data were identified using the *FindMarkers* function from Seurat based on overlapping cell types. Mitochondrial, ribosomal and protocol differential genes were then removed from the reference data. To evaluate the effect of gene expression-based filtering of the reference data on the performance of deconvolution algorithms, we initially calculated the per cell type percentage of expressing cells (with expression intensity >0) for each gene in the single-cell reference data. Then, a threshold was applied on percentage of expressing cells to identify genes with at least one cell type passing the threshold. Six thresholds were evaluated in total: 0%, 5%, 10%, 20%, 30% and 40%. Predictions were made for all eight cell types in the reference data. Since only three major cell types were annotated from CaseViewer (epithelial cells, stroma, adipocytes), we combined deconvolution predictions for fibroblasts, pericytes and endothelial cells into the ‘stomal’ category, in alignment with definitions from a recent study (39). Finally, predictions from different deconvolution algorithms were compared with CaseViewer estimations using metrics like RMSE and SCC.

### Evaluation on GTEx breast FFzn samples

Bulk FFzn RNA-seq data for 129 female breast tissue were retrieved from GTEx (40). Data were normalized to TPM and genes expressing at low levels (average TPM < 4) were excluded. Genes relating to mitochondria, ribosomes and protocol differentials were also removed from the integrated single-cell reference data. The reference data were further filtered to exclude genes with <20% of expressing cells per cell type. Data were then normalized using the *LogNormalize* function from Seurat (38). Scaden was used to predict cell type proportions across the 129 breast samples. We extracted annotations for fat percentage and manually parsed this information from the pathology notes of the GTEx project. A total of 96 subjects with available pathology annotations for fatty tissue were identified. We separated those samples into two groups based on annotated fat percentages (>50% versus < 50%). Finally, Scaden-predicted proportions of adipocytes were compared between the two groups using the Wilcoxon rank sum test.

## Results

### Construction of integrated scRNA-seq reference data

To facilitate deconvolution evaluation, we curated two independent public breast tissue single-cell datasets that employed different protocols, scRNA-seq and snRNA-seq. After thorough QC, normalization and data integration procedures, we observed overall high concordance between cell types from the two data sources (Supplementary Figure S1). snRNA-seq was more sensitive in capturing adipocytes, while scRNA-seq was more sensitive in detecting lymphocytes in breast tissue. These observations align with recent findings from a study on lung and skin tissues (34). Overall, we have constructed a comprehensive single-cell reference dataset for female breast tissue, tailored for cell type deconvolution for bulk RNA-seq data.

### Benchmark of deconvolution methods using simulated experiments

**Figure 2.**
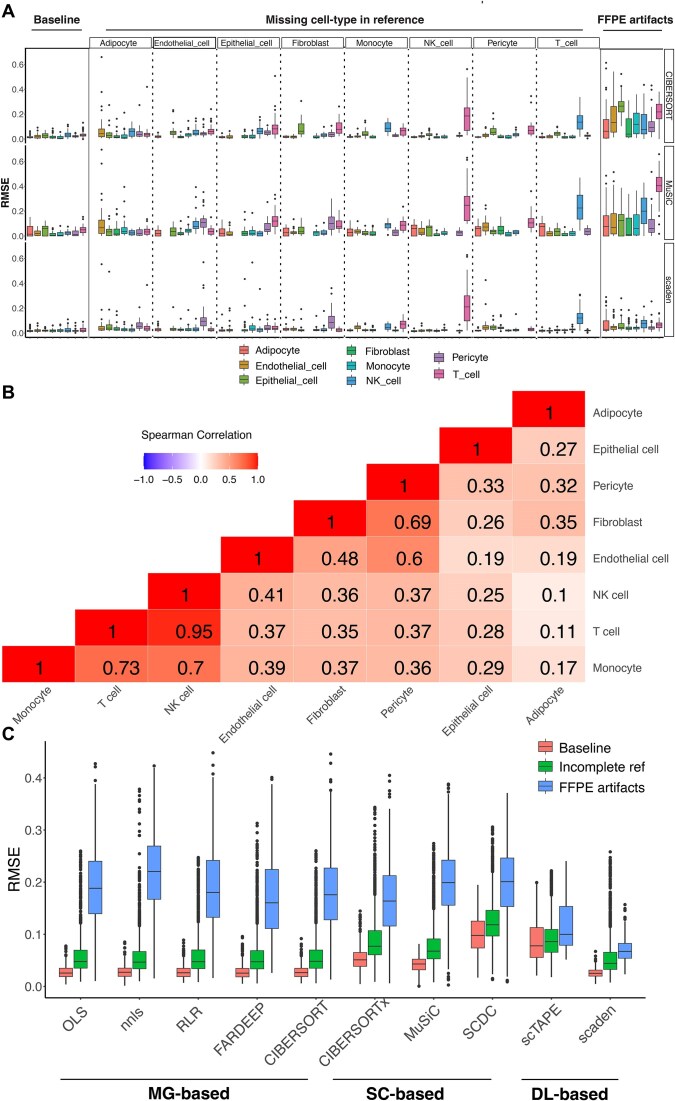
Comparative analysis of deconvolution methods using simulated experiments. The benchmarking process entailed three distinct simulation scenarios: (i) baseline simulations; (ii) incomplete references, wherein specific cell types were omitted from the reference data; and (iii) FFPE artifact simulation, grounded on our proprietary FFPE–FFzn sample pair data. (**A**) RMSE evaluations for three representative methods from each category: MG-based, SC-based and DL-based. (**B**) Heatmap showing cell type correlations based on scRNA-seq reference data. (**C**) Aggregated RMSE metrics spanning all three experimental situations for all deconvolution methods.

To critically assess 10 distinct deconvolution methods, we generated simulated experiments categorized into three main tests: (i) baseline (ideal situation); (ii) incomplete references, where we randomly removed a certain cell type from the reference data; and (iii) FFPE artifacts, where we simulated FFPE artifacts based on in-house FFPE–FFzn sample pairs (Supplementary Figure S2). Figure 2A shows performances in terms of RMSE of one selected deconvolution method from each of the three categories (MG-based, SC-based and DL-based). Under optimal conditions, most methods achieved similar performance (Figure 2A and Supplementary Figure S3, median RMSE range: 0.019–0.04) with similar bias (Supplementary Figure S4, median absolute bias range: 0.01–0.038) and variance (Supplementary Figure S5, median variance range: 5.3E−5 to 2.2E−4). SCDC and scTAPE performed slightly worse than the other methods (median RMSE for SCDC/scTAPE: 0.028/0.04). Following exclusion of a cell type from the reference data, we noted an amplification in prediction errors that were not evenly distributed across the remaining cell types (Figure 2A). Removing a cell type elevated prediction errors for transcriptomically similar cell types (Figure 2B). For instance, the removal of NK cells dramatically increased the prediction error for T cells with little effect on the rest of cell types (median RMSE for T cells/other cells: 0.211/0.02). This pattern can be rationalized by the fact that there is a high correlation between T cells and NK cells in terms of transcriptomic activity (Figure 2B, SCC = 0.95). The predominant source of prediction errors is attributed to bias rather than variance, indicating the increase of systematic error caused by incomplete reference datasets (Supplementary Figures S4 and S5, median bias^2^/median variance: 4.8E−4/2.3E−4). Scaden displayed superior performance with good control of bias across the majority of cell categories. Single-cell reference-based methods tend to be more sensitive to cell type removal experiments and displayed worse performance compared to other methods (Figure 2C).

With the simulation experiments including FFPE artifacts, we observed elevated prediction errors with similar bias/variance contributions across most methods (Supplementary Figures S4 and S5, median bias^2^/median variance: 2.5E−3/2.6E−3). Scaden and scTAPE displayed excellent control of variance and better control of bias compared to other methods (median absolute bias: 0.02 versus 0.1). Marker gene-based methods exhibited higher prediction errors, especially for epithelial cells and T cells (median RMSE for epithelial cells/T cells/other cells: 0.29/0.21/0.11). Single-cell reference-based methods manifested higher prediction errors with T cells and NK cells (median RMSE for T cells/NK cells/other cells: 0.36/0.19/0.07). Overall, Scaden showed better performance across all three simulation experiments, and was especially robust to FFPE artifacts. scTAPE was the second-best method when FFPE artifacts were introduced but was sensitive to cell type removal.

### Benchmark of deconvolution methods using BBD FFPE samples

**Figure 3.**
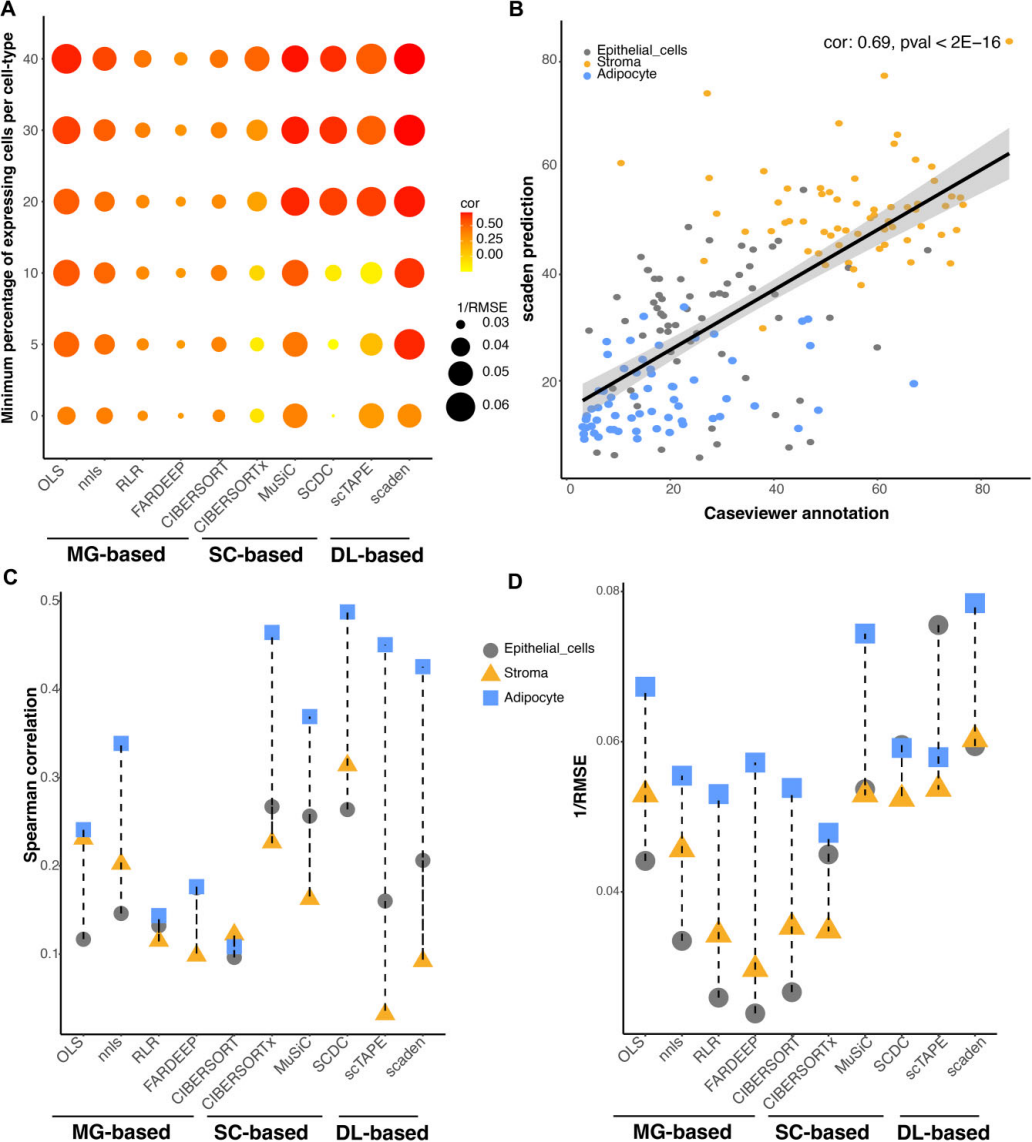
Performance assessment of deconvolution methods on real data from BBD FFPE samples. Predictions from each deconvolution methods were compared with CaseViewer annotations. (**A**) The analysis of gene-filtering stringency on the overall performance of deconvolution methods for all three cell types. Dot color indicates SCC with CaseViewer annotation. Dot size indicates the inverse of RMSE. The *Y*-axis represents the minimum requisite percentage of expressing cells per cell type. For example, a value of ‘0’ means no gene filtering was applied, while ‘40′ indicates that at least one cell type must have over 40% of its cells expressing the gene. (**B**) A scatterplot juxtaposing CaseViewer annotation and predictions from Scaden at 20% cutoff. Colors indicate the three major cell types evaluated. SCC and associated *P*-value were annotated at the top right. (**C**) Insight into each cell type’s correlation performance with CaseViewer estimates across deconvolution methods. Color and shape of the dot indicate SCC values for individual cell types. (**D**) Precision of cell type in terms of the inverse of RMSE. Color and shape of the dot indicate 1/RMSE values for individual cell types.

We quantified the composition percentages of epithelial, adipose and stroma components across all 62 BBD samples using H&E images and CaseViewer, as detailed in the ‘Materials and methods’ section (Supplementary Figure S6). RNA-seq data from these 62 BBD FFPE samples underwent QC and normalization, and then were subjected to the 10 deconvolution methods. Genes with relatively low expression intensity were more affected by the FFPE procedure than those with higher intensity, as evidenced by more pronounced variations when compared with paired FFzn samples (7) (Supplementary Figure S2). To evaluate the effect of our pre-filtering strategies on the performance of the deconvolution methods, we instituted a step to exclude genes with minimal expression from the single-cell reference data (Supplementary Figure S7). Figure 3A shows the aggregate SCCs and RMSEs across all cell categories. All methods tend to have increased performance with increased filtering stringency on gene expression (median RMSE 0%/40%: 28.1/19.2; median SCC 0%/40%: 0.33/0.57). Both MuSiC and Scaden are relatively resilient against the extent of filtering, whereas SCDC and scTAPE only start to perform well with moderate filtering stringency (20%). The performance of most methods tends to plateau when the filtering stringency reaches 20%. Conversely, RLR, FARDEEP and CIBERSORT underperform even with intense gene expression filtering. Figure 3B shows the relationship between CaseViewer annotation and Scaden predictions across all cell types. Overall, Scaden and OLS are top-performing methods in terms of both RMSE and SCC. We then evaluated the performance of different deconvolution methods specific to each cell type. As shown in Figure 3C and D and Supplementary Figures S8 and S9, all methods showed decreased SCC values when evaluated at the cell type level (41). Moreover, the efficiency varies between cell types (median SCC at 20% filtering stringency: epithelial cells/stroma/adipocytes: 0.17/0.14/0.35). Similar findings were reported from a previous study involving FFzn samples (17). Due to the lack of capability to directly quantify immune cell populations using PatternQuant from CaseViewer, we performed IHC staining for CD45, a marker indicative of immune cells, using H&E slides for 11 randomly selected samples. Percentage of CD45-positive cells was then quantified using CaseViewer. As shown in Supplementary Figure S10, we observed a strong correlation between deconvolution-predicted immune cell proportions (T cells, monocytes and NK cells) from Scaden, and percentage of CD45-positive cells quantified from CaseViewer (SCC: 0.64; *P*-value: 0.03). Those results suggest that the deconvolution strategies can be readily applied to immune cell populations as well. We then conducted further analyses to investigate the influence of FFPE block age on the performance of various deconvolution methods, as illustrated in Supplementary Figure S11. Notably, the performance of marker gene-based methods decreased with older FFPE blocks. In contrast, for SC-based and DL-based methods, there is no discernible association between deconvolution performance, especially in terms of RMSE, and the age of the FFPE blocks. This suggests the robustness of those methods against block age. Overall, SCDC and Scaden are the top-performing methods, gauged by SCC and RMSE, respectively, indicating the potential utility of these methods for inferring cell type-specific gene expression levels. Despite the broader satisfactory performances, the results indicate that predictions at the individual cell type tier may still require enhancement.

### Evaluation of deconvolution methods using FFzn samples from the GTEx breast tissue collection

To verify the applicability of our optimized strategies to FFzn samples, we applied the top-performing method, Scaden, to data derived from 129 FFzn breast samples from GTEx female subjects. The single-cell reference data were filtered to remove mitochondrial, ribosomal and protocol differential genes, as well as genes with minimal expression, measured at 20% or below, and those with a TPM below 4 in the bulk data. We observed a wide range of cell type compositions across the samples (Supplementary Figure S12A). For instance, the range of epithelial proportions spanned from 0.4% to 44%. We then classified these samples into two groups based on pathology annotations for fat content: samples with >50% fat tissue versus samples with less fat tissue. It should be noted that pathology annotations determine the area occupied by a particular cell type, while gene expression-based deconvolution predictions focus on the cellularity of the cell type. While the foundational concepts behind these measurement methods differ, we still expect to see a consistent trend between the estimations. As shown in Supplementary Figure S12B, samples with >50% fat tissue indeed have significantly more predicted proportion of adipocytes, compared to samples with <50% fat tissue. Those results highlight the applicability of our optimized deconvolution strategies and the heterogeneity of cell type proportions within GTEx breast samples. Future studies leveraging GTEx data will benefit from accounting for variations in cell type proportions for their analysis.

### Streamlined framework for reproducible simulation experiments and downstream analyses

**Figure 4.**
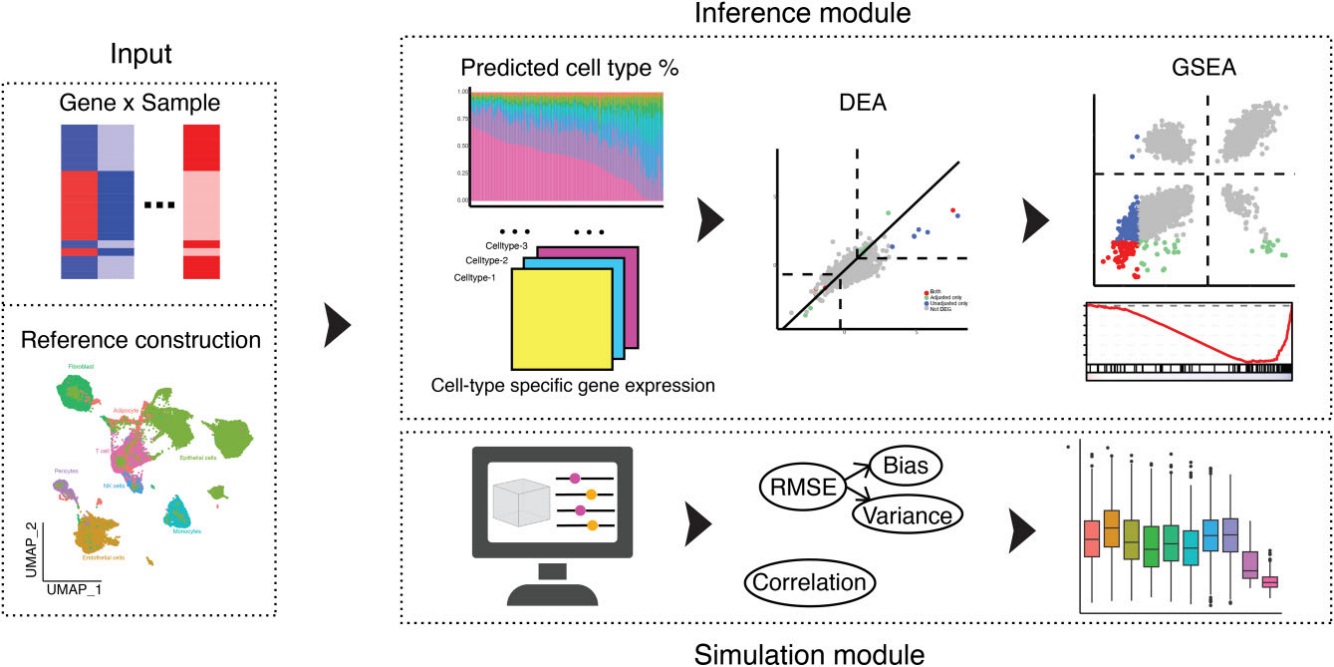
Overview of SCdeconR. SCdeconR provide functionalities to (i) assemble integrated scRNA-seq reference data, (ii) facilitate the creation of simulated experiments for evaluation of deconvolution methods and (iii) perform cell type deconvolution and downstream analyses, including DEA and GSEA. For those interested in utilizing or exploring SCdeconR, it is currently accessible from CRAN at https://CRAN.R-project.org/package=SCdeconR and GitHub at https://github.com/Liuy12/SCdeconR (created with BioRender.com).

While this study focused on deconvolution of breast tissue, we anticipate that our analytical workflow and simulation framework can be adapted effectively to other tissue types. To facilitate reproducible research and future studies involving FFPE samples from other tissue types, we built an R package for deconvolution-related analyses (Figure 4). Named SCdeconR, this package aims to provide a streamlined and flexible toolkit ranging from deconvolution of bulk RNA-seq data to complex DEA and GSEA. SCdeconR is structured into three modules: (i) the input module, crafted to compile reference scRNA-seq data for different tissue types; (ii) the simulation module, designed to create simulation experiments for evaluation purposes; and (iii) the inference module, dedicated to deconvolution prediction and advanced analyses like downstream DEA and GSEA. To further enhance its usability, SCdeconR also provides several visualization options, enabling users to assess the impact of cell proportion adjustments on differential expression and pathway analyses. Those interested can access SCdeconR on CRAN (https://CRAN.R-project.org/package=SCdeconR) and GitHub (https://github.com/Liuy12/SCdeconR).

## Discussion

In this study, we endeavored to shed light on the complexity of breast tissue deconvolution methods. Initially, we assembled comprehensive single-cell reference data for normal breast tissue by consolidating data from two distinct studies. Harnessing the strengths of both scRNA-seq and snRNA-seq protocols, we pinpointed the eight major cell types in female breast tissue. We then conducted rigorous simulation experiments to assess different deconvolution methods across (i) baseline efficacy, (ii) effects of incomplete reference data and (iii) resilience against FFPE artifacts. At the baseline level, all methods achieved good results. During cell type removal tests, Scaden emerged as the best performer, demonstrating exceptional bias control. In simulations examining the effects of FFPE artifacts, Scaden and scTAPE outperformed their counterparts in bias and variance management. We then further evaluated all methods against imaging-based annotations from 62 BBD FFPE samples. A consistent observation was the enhancement in methodological performance with increased filtering stringency of gene expression levels on the reference data. In across cell type evaluations, Scaden and OLS emerged as the top-performing methods. At the individual cell type level, Scaden and SCDC were the top-performing methods based on RMSE and SCC, respectively. We evaluated the top-performing method, Scaden, on a cohort of FFzn samples from the GTEx breast tissue collection. Notably, the proportion of adipocytes predicted by Scaden was significantly associated with pathology evaluations. Finally, we built an R package SCdeconR to streamline future cell type deconvolution-related analyses.

Previous studies evaluating deconvolution methods primarily employed RMSE or Pearson correlation metrics and centered their analyses on across cell type performances (11,23). We introduced an innovative simulation design that not only examined conventional metrics, but also dissected errors to uncover their bias and variance components. This approach provided a deeper comprehension of each algorithm’s strengths and weaknesses. In addition to simulation experiments, our methodology also incorporated imaging-based digital annotations of cell types, sourced from H&E slides of breast tissue FFPE samples. This allowed for a more complete examination of both across cell type and within cell type performances, shedding light on inherent challenges and offering recommendations for real-data predictions at the within cell type level. To facilitate future research endeavors, we have encapsulated our combined methodologies into an R package, SCdeconR. This tool integrates a simulation framework with an inference module for downstream DEA and GSEA.

There are several limitations to our study that should be acknowledged. To benchmark deconvolution methods using real data, we relied on imaging-based cell type annotations derived from a fusion of PatternQuant and CellQuant algorithms from CaseViewer. These methods do not classify immune populations; consequently, we have identified and profiled three primary cell types: epithelial cells, stroma and adipocytes. In the context of each H&E slide, immune cells are categorized into one of these three categories based on their imaging characteristics. Cell type proportions are determined by the ratio of nuclei specific to a cell type to the total detected nuclei, a method potentially influenced by the categorization of immune cells. To evaluate deconvolution predictions on immune cell population, we performed IHC staining of CD45, a marker indicative of immune cells, and observed that the proportion of CD45-positive cells was generally higher than that of deconvolution-predicted immune cells (T cells, monocytes and NK cells). The differences in value ranges between the two metrics may stem from the fact that composition of CD45-positive cells encompasses not solely T cells, monocytes and NK cells, but also additional cellular subtypes originating from myeloid and lymphoid lineages (42). For future study, we advocate conducting scRNA-seq experiments directly on FFPE samples to enhance the precision in quantifying immune cell proportions. In our evaluation of the GTEx breast tissue cohort, we had to bifurcate samples into two groups due to the lack of consistent format for pathology annotations of fat tissue. Notably, while pathology annotations gauge fat-based adipose tissue area, deconvolution-derived adipocyte populations align more with the actual adipocyte cell count. This distinction emphasizes that these two metrics should not be directly compared. Our analysis also unveiled a stark drop in performance for within cell type evaluations in comparison to across cell type evaluations, with the disparity being particularly prominent for epithelial and stroma components. This variance might be attributed to the intrinsic heterogeneity of those components in the single-cell reference data (33). Finally, the strategies we defined here are based on breast tissue and might not be readily applicable to other tissue types. To address this, we have ensured that our simulation framework within SCdeconR is sufficiently versatile to support future evaluations across different tissue types. As we advance our understanding of cell type deconvolution, future studies should prioritize expansion and refinement of existing datasets, focusing on underrepresented cell types such as immune populations. The insights obtained from our study, combined with the versatility of tools like SCdeconR, set the stage for more nuanced and comprehensive analyses across a broader spectrum of tissue types, thereby deepening our grasp of cellular complexities and their implications in health and disease.

## Supplementary Material

lqae098_Supplemental_File

## Data Availability

scRNA-seq data for Bhat-Nakshatri’s group were downloaded from GEO (GSE164898). Raw data (.h5ad) for snRNA-seq data were downloaded from the GTEx single-cell portal (https://gtexportal.org/home/datasets, V9). RNA-seq data for our BBD cohort can be downloaded from GEO (GSE244325). All other datasets used in this study can be downloaded from Zenodo (https://zenodo.org/records/11374072).
