# Supplementary material for: Evaluating cell type deconvolution in FFPE breast tissue: application to benign breast disease
Source: NAR Genom Bioinform. 2024 Aug 6;6(3):lqae098. doi: 10.1093/nargab/lqae098 (PMC11952925; doi:10.1093/nargab/lqae098)
Supplement: lqae098_Supplemental_File [file lqae098_supplemental_file.pdf]

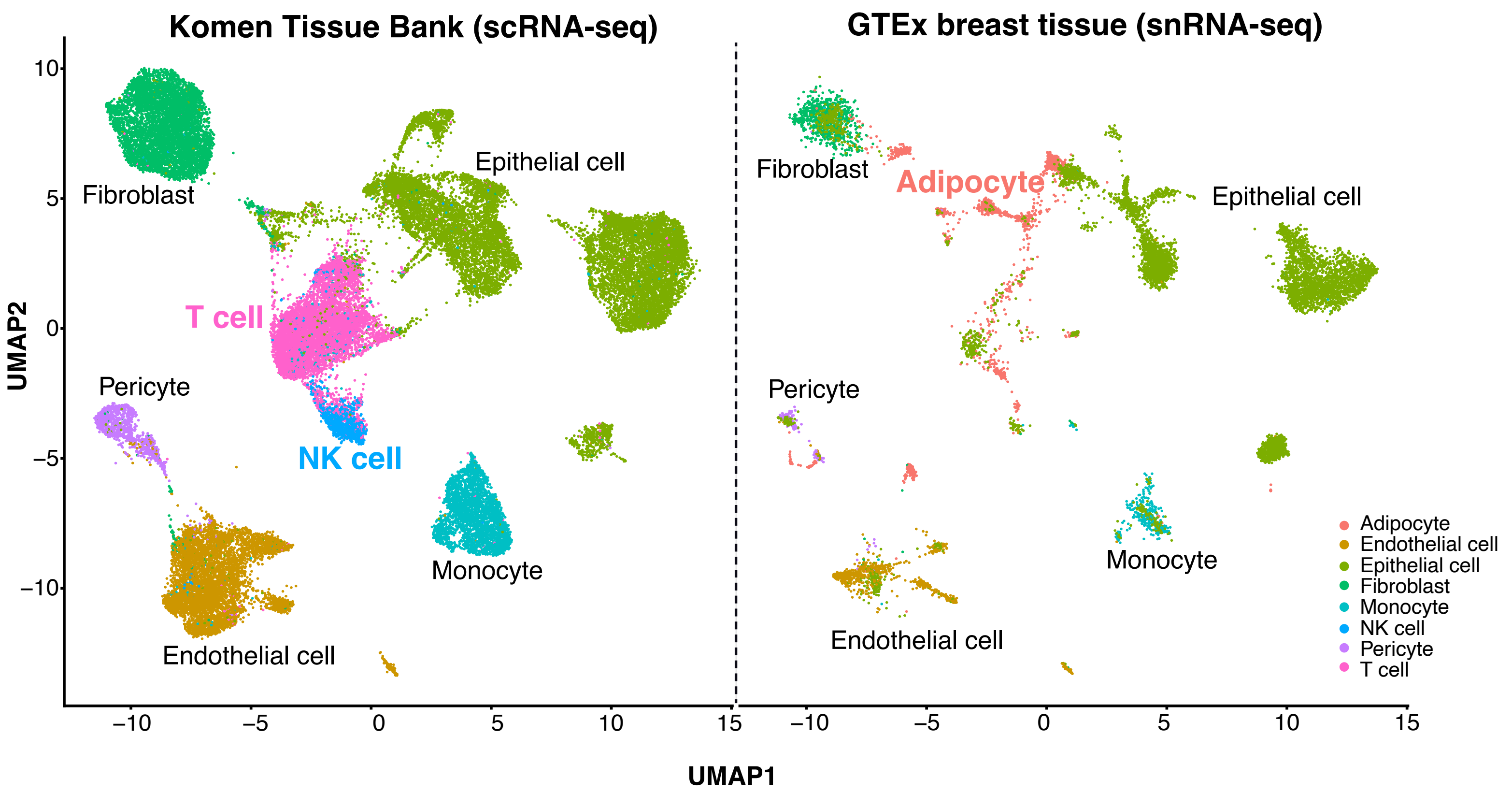

Figure S1. Construction of integrated breast tissue single cell reference using public data from two resources (scRNA-seq data using samples from Komen Tissue Bank, snRNA-seq data for GTEx breast tissue). Overall high concordance were observed among cell types between the two data sources. snRNA-seq was more sensitive in capturing adipocytes (highlighted in red, right panel), while scRNA-seq was more sensitive in detecting lymphocytes in breast tissue (highlighted in pink and blue, left panel).

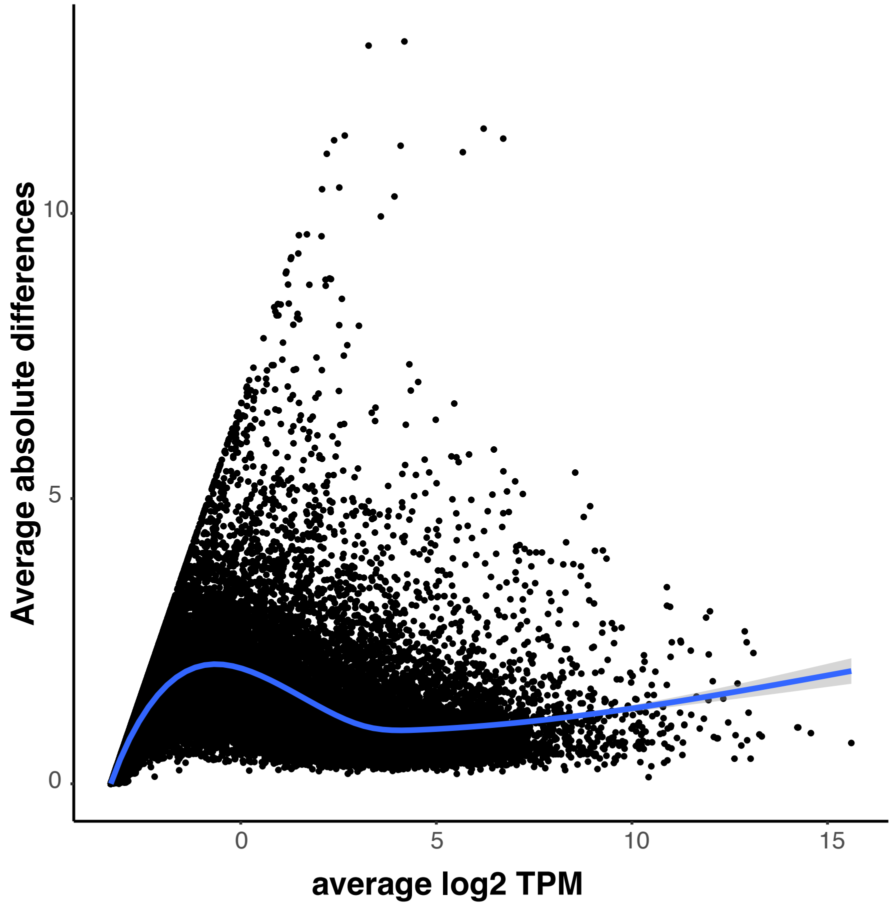

Figure S2. Relationship between gene expression levels in TPM and FFPE induced artifacts. Scatter plot between average log2 TPM and average absolute differences between 7 paired FFPE-FFzn samples. The relationship was fitted using a generalized additive model, and was used to introduce FFPE artifacts for simulation experiment.

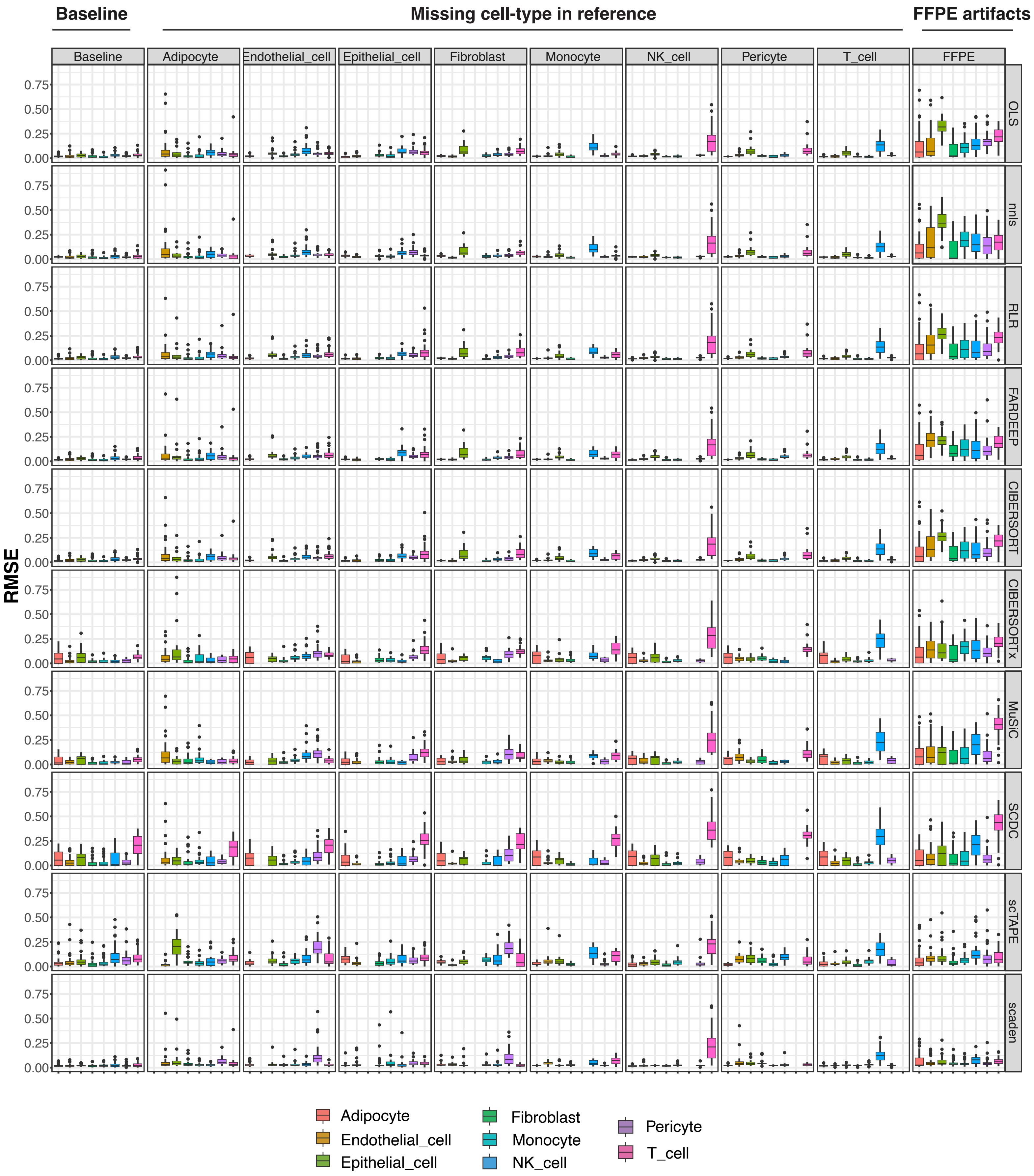

Figure S3. Performance of all methods in terms of RMSE across the three types of simulation experiments: 1. baseline; 2. Incomplete reference, where we randomly removed a certain cell type from the reference data; 3. FFPE artifacts, where we simulated FFPE artifacts based on in-house FFPE-Fresh Frozen sample pairs.

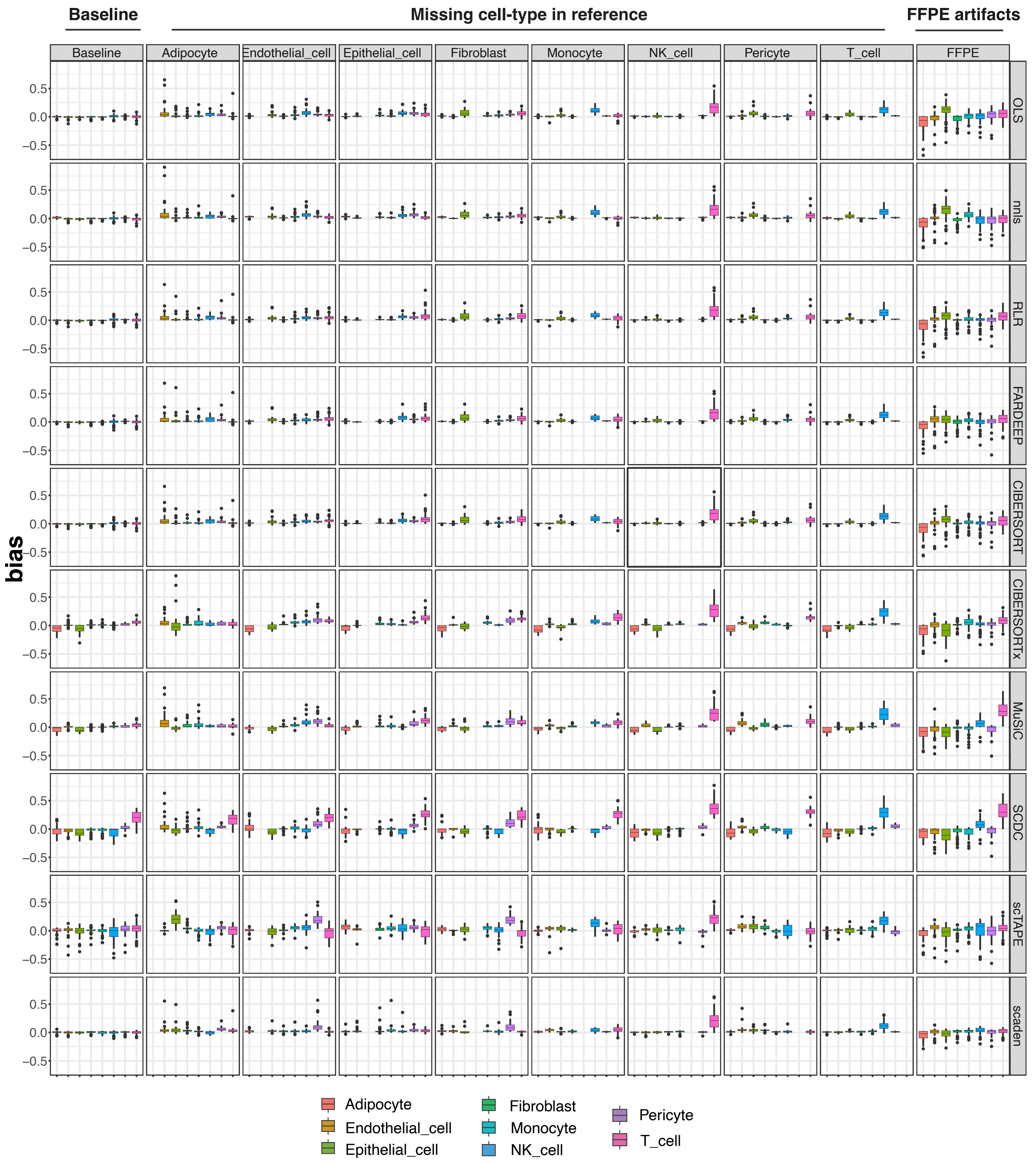

Figure S4. Performance of all methods in terms of bias across the three types of simulation experiments: 1. baseline; 2. Incomplete reference, where we randomly removed a certain cell type from the reference data; 3. FFPE artifacts, where we simulated FFPE artifacts based on in-house FFPE-Fresh Frozen sample pairs.

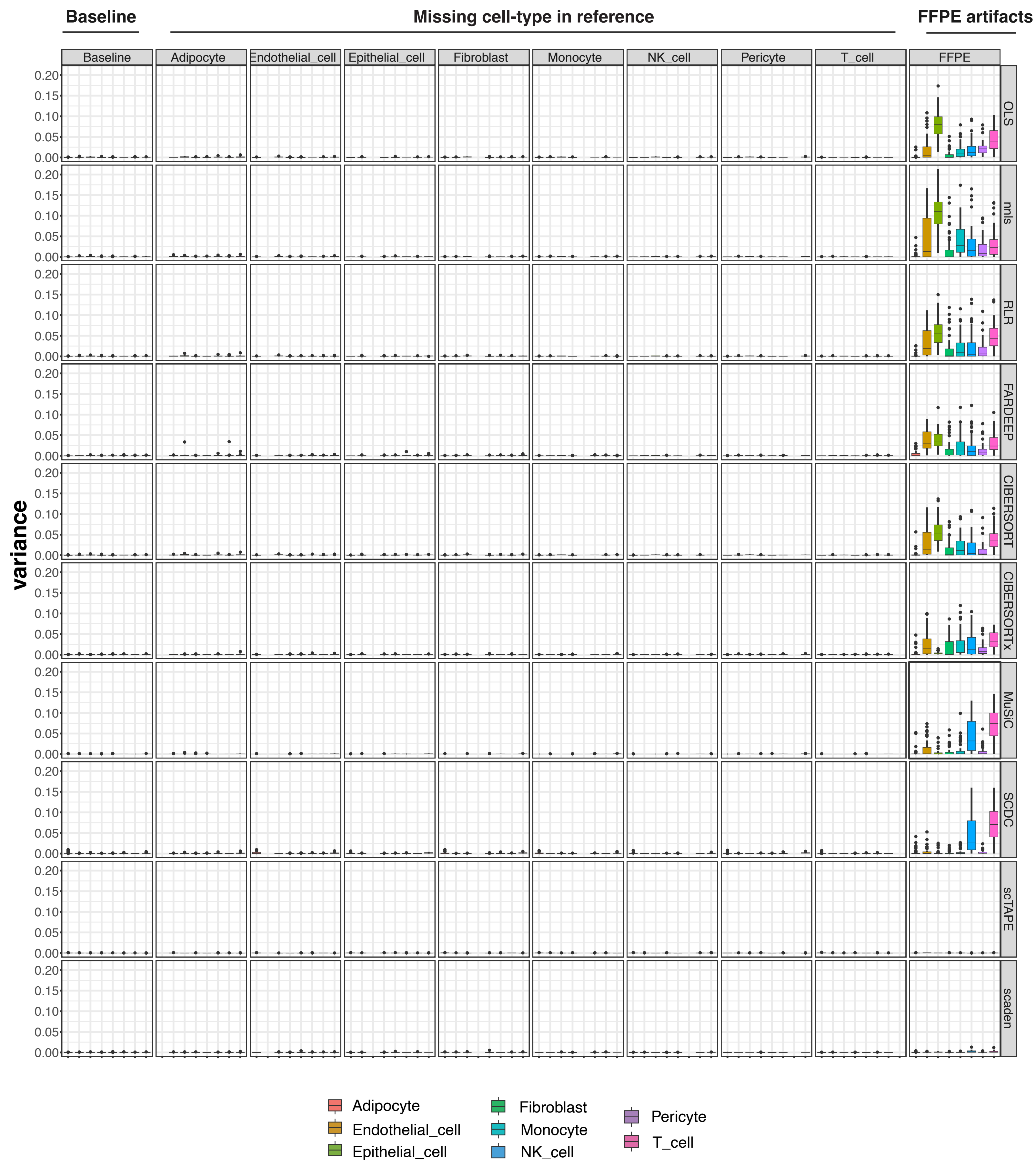

Figure S5. Performance of all methods in terms of variance across the three types of simulation experiments: 1. baseline; 2. Incomplete reference, where we randomly removed a certain cell type from the reference data; 3. FFPE artifacts, where we simulated FFPE artifacts based on in-house FFPE-Fresh Frozen sample pairs.

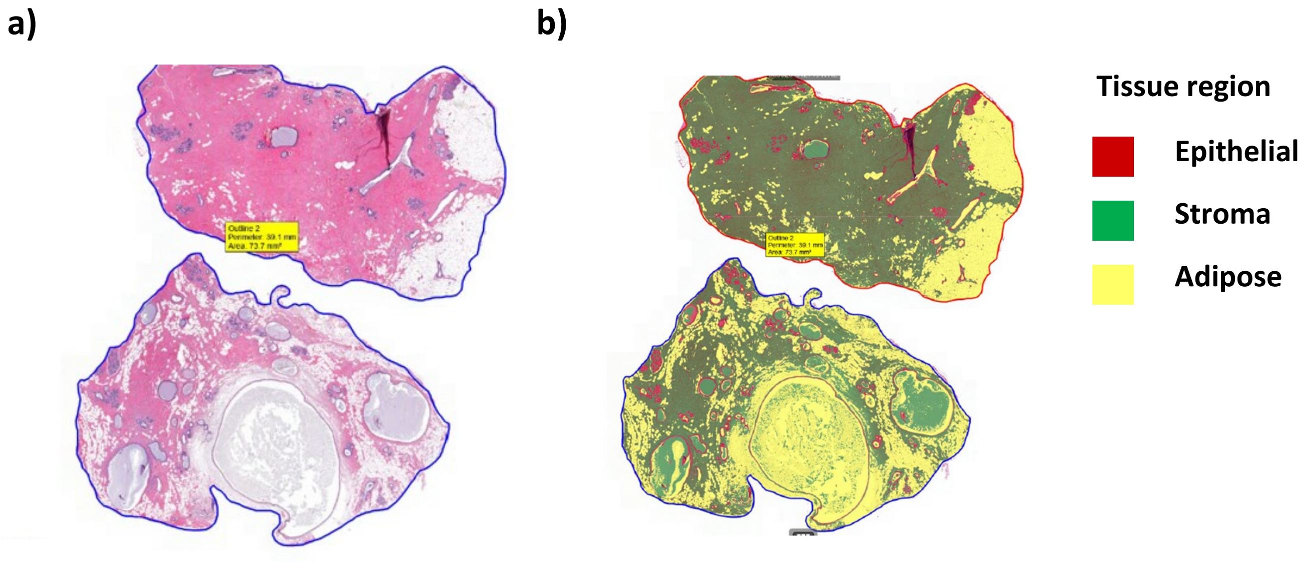

Figure S6. Example segmentation and nuclei quantification of digitized H/E Slide using CaseViewer software. After training the machine-learning model using example H/E slides with segmentations for the epithelial, stromal, and adipose tissue regions, CaseViewer's Pattern Quant module segments new H/E slides into those tissue regions and the CellQuant module can identify cellular nuclei and determine the number of cellular nuclei within each tissue region. a) An example H/E slide. b) Resulting segmentation for each tissue region (Epithelial, Adipose, and Stroma) from CaseViewer. Yellow text box denotes the estimates of perimeter and area.

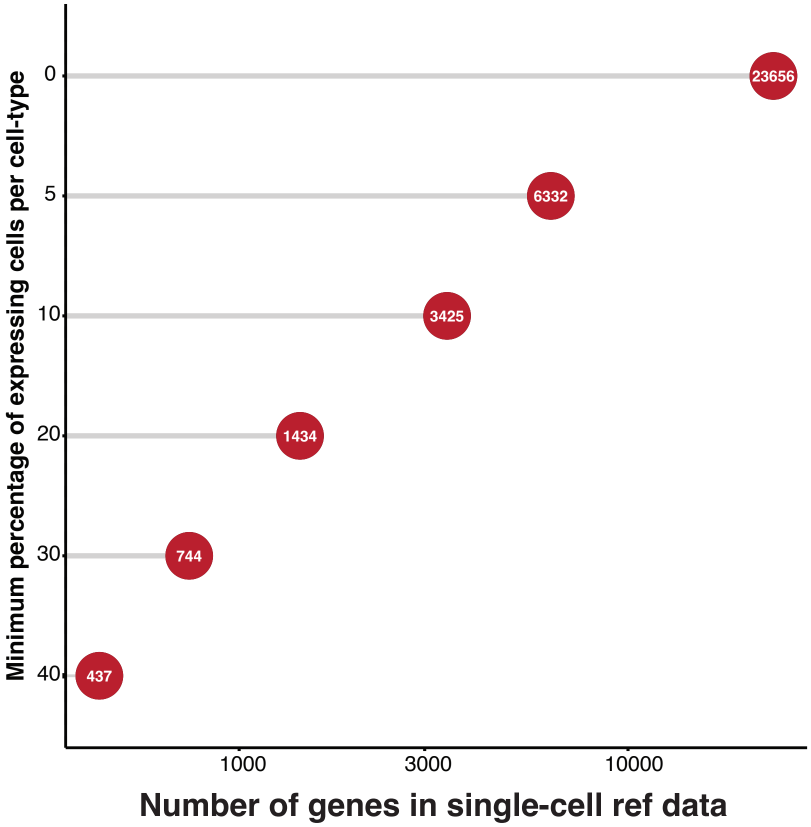

Figure S7. Number of genes in the scRNA-seq reference data that passed filtering for each threshold (0%, 5%, 10%, 20%, 30%, 40%);

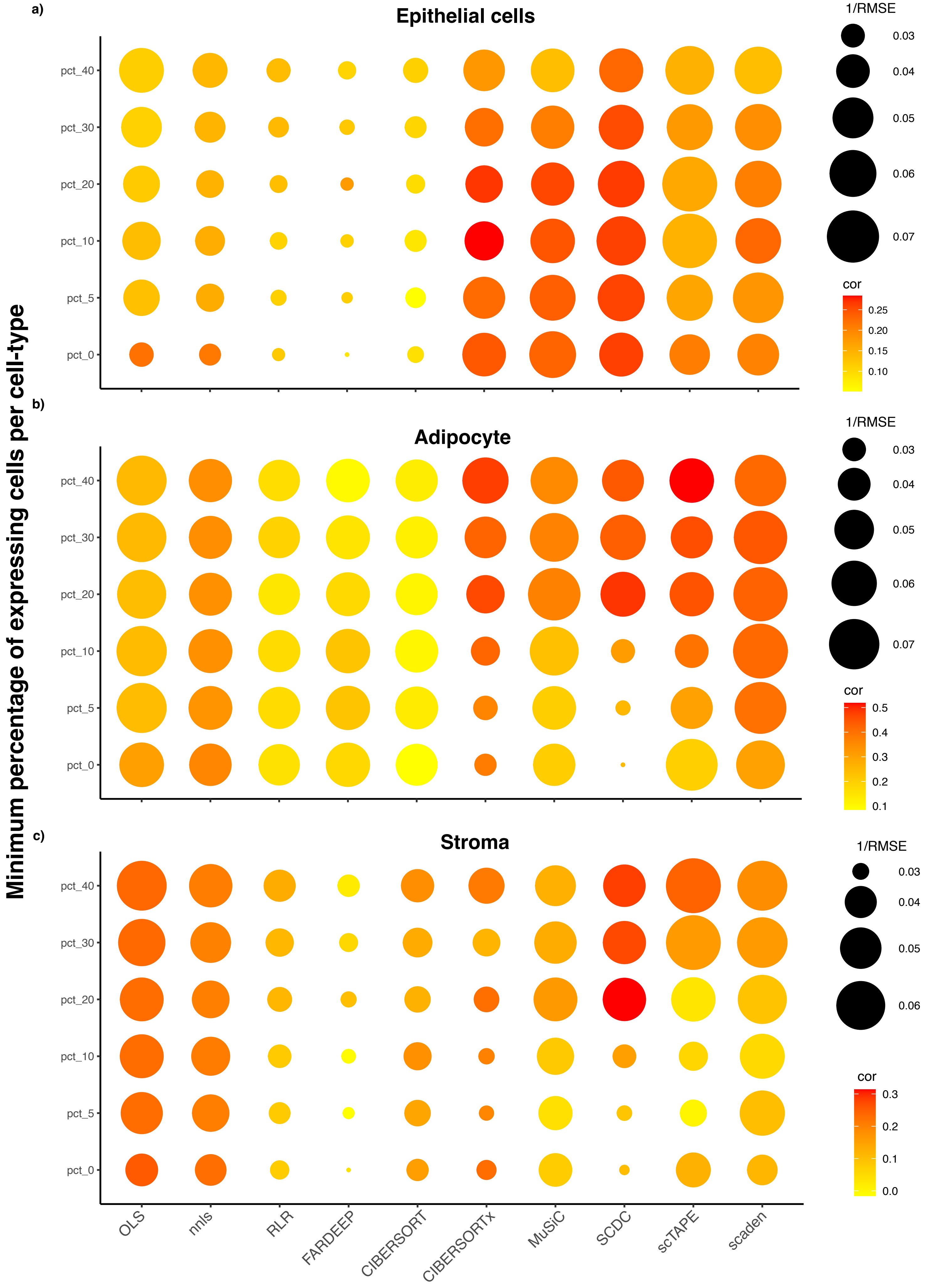

Figure S8. Per cell-type performances of deconvolution methods in terms of 1/RMSE and Spearman correlation.

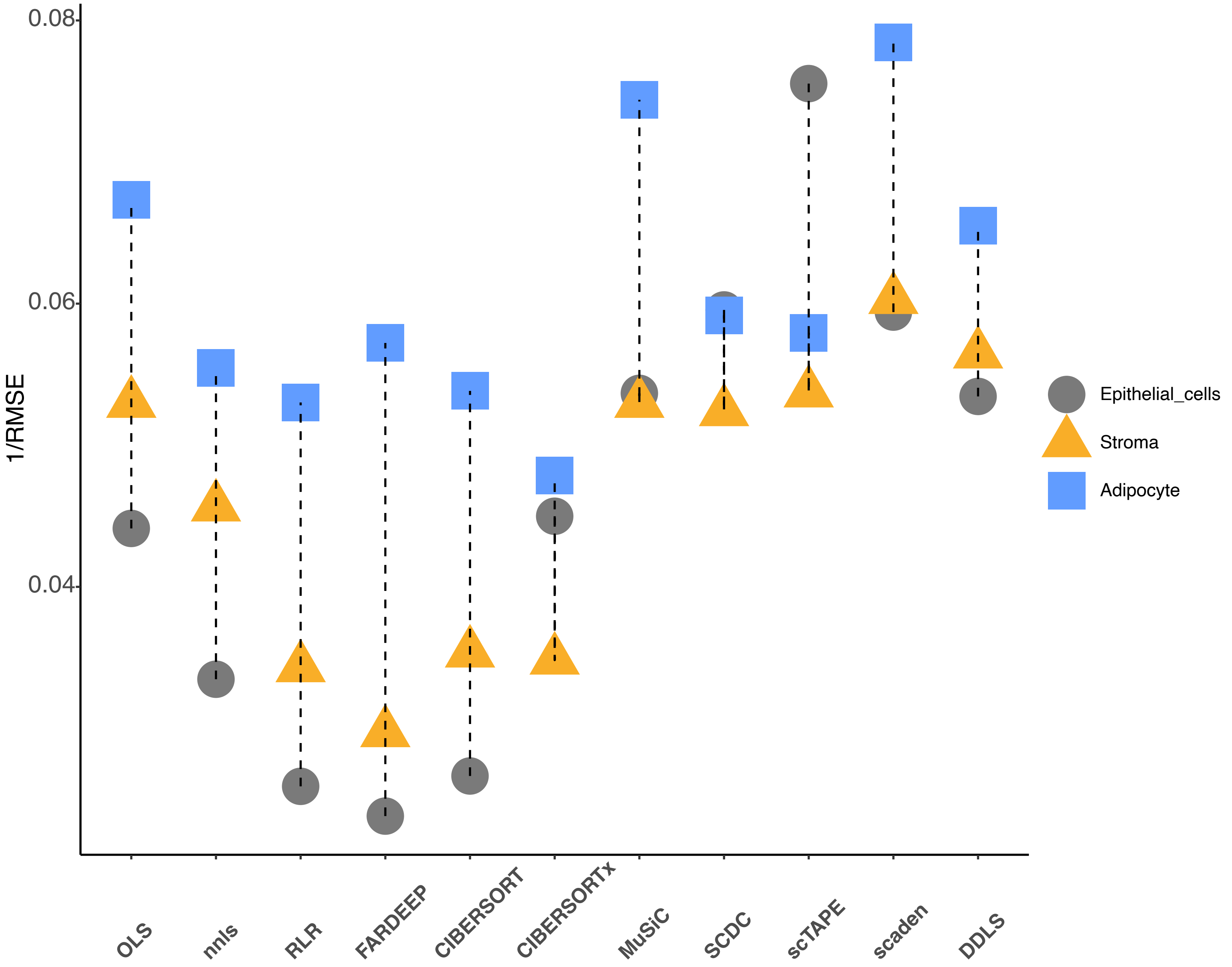

Figure S9. Precision of cell type in terms of the inverse of RMSE. Color and shape of the dot indicate 1/RMSE values for individual cell types. digitalDLSorteR (DDLS) was included to demonstrate the flexibility of SCdeconR for including additional deconvolution algorithms.

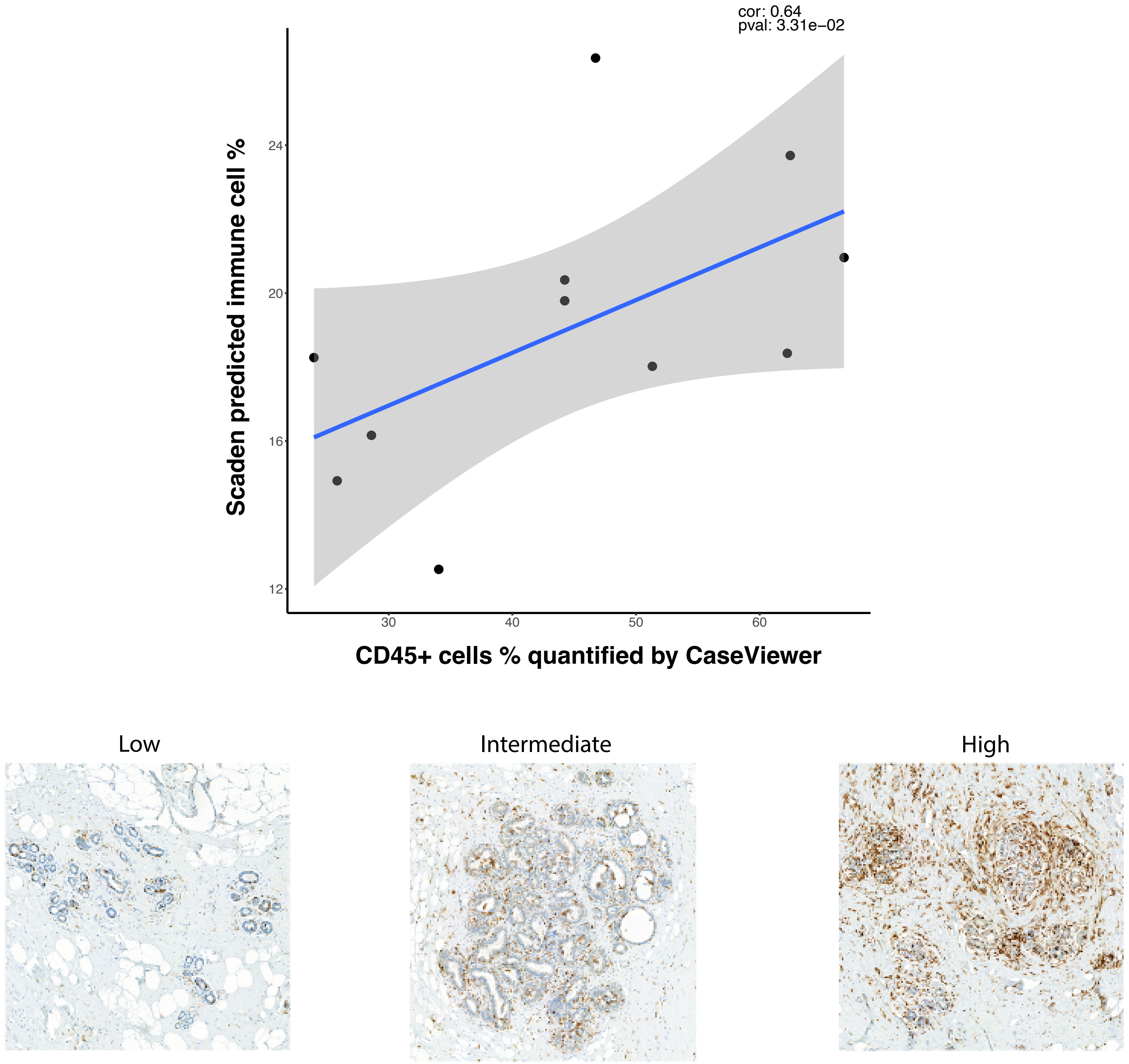

Figure S10. Scatter plot between CD45+ cells % quantified by CaseViewer and Scaden predicted immune cell %. Three representative examples were shown (5X magnification) based on proportion of CD45+ cells (low, intermediate, and high).

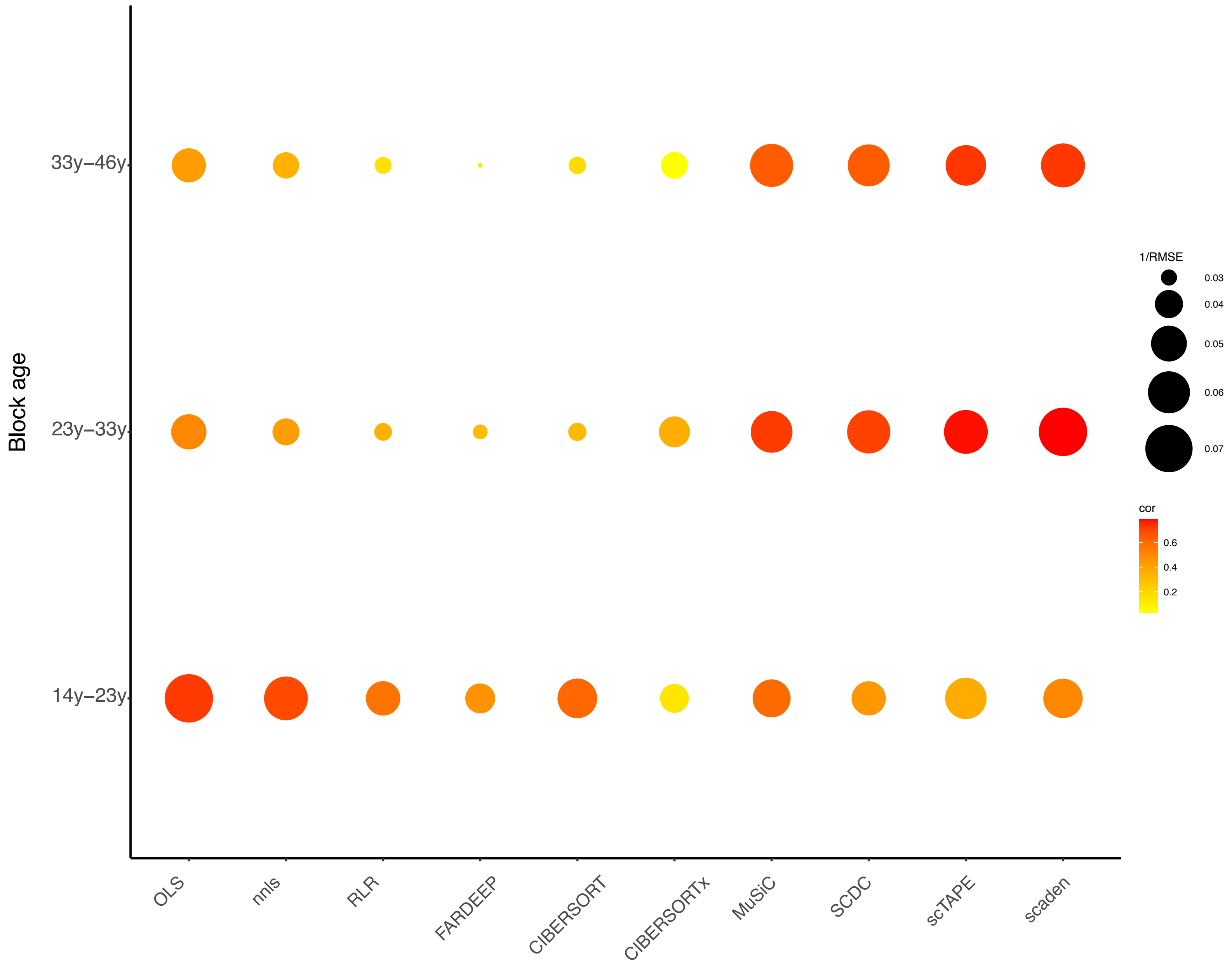

Figure S11. Performance of deconvolution algorithms grouped by the age of FFPE blocks: 14-23 years, 23-33 years, and 33-46 years

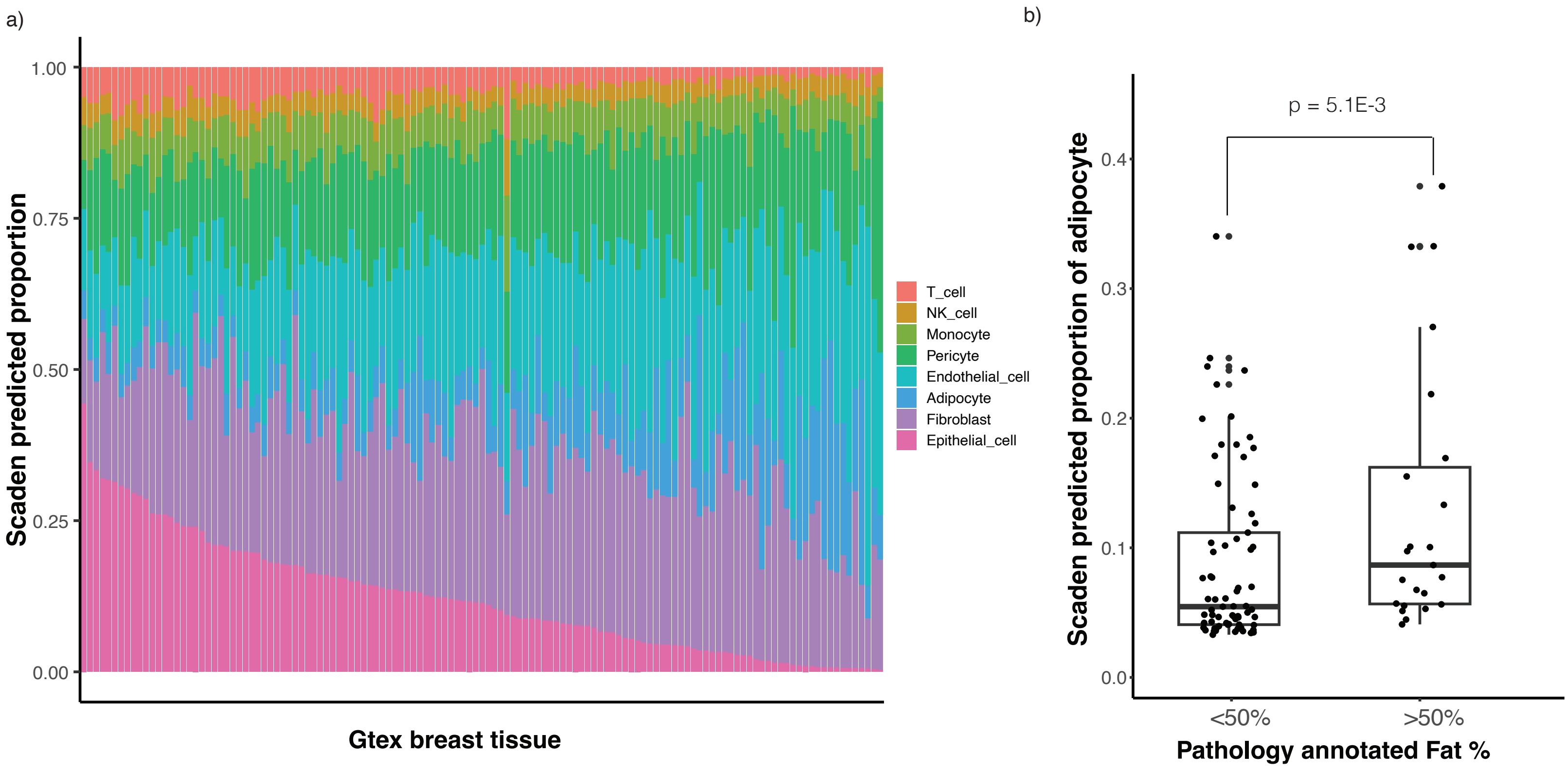

Figure S12. Evaluate the performance of Scaden on GTEx FFzn samples. a) Scaden predicted cell type proportions for all 292 female breast samples; b) Comparison between Scaden predicted proportions (adipocytes) and pathology annotated fat percentage (<50 indicates smaller than 50% of fat tissue, >50 indicates larger than 50% fat tissue). P values from Wilcoxon rank sum test were annotated for the comparison.
